# 黄芪桂枝五物汤治疗非小细胞肺癌软脑膜转移患者培美曲塞鞘内化疗神经毒性的随机对照研究

**DOI:** 10.3779/j.issn.1009-3419.2026.102.02

**Published:** 2026-02-20

**Authors:** Huiying LI, Xin CHEN, Yu XIE, Cheng JIANG, Yongjuan LIN, Tingting YU, Zhenyu YIN

**Affiliations:** ^1^210008 南京，南京大学医学院附属鼓楼医院老年肿瘤科; ^1^Department of Geriatric Oncology, Nanjing Drum Tower Hospital, Affiliated Hospital of Medical School, Nanjing University, Nanjing 210008, China; ^2^210008 南京，南京中医药大学附属鼓楼医院老年肿瘤科; ^2^Department of Geriatric Oncology, Nanjing Drum Tower Hospital, Clinical College of Nanjing University of Chinese Medicine, Nanjing 210008, China

**Keywords:** 肺肿瘤, 软脑膜转移, 培美曲塞, 神经毒性, 黄芪桂枝五物汤, Lung neoplasms, Leptomeningeal metastases, Pemetrexed, Neurotoxicity, Huangqi Guizhi Wuwu decoction

## Abstract

**背景与目的:**

软脑膜转移（leptomeningeal metastasis, LM）是非小细胞肺癌（non-small cell lung cancer, NSCLC）的严重并发症，培美曲塞鞘内化疗能有效延长患者生存，但较高的神经毒性发生率已成为限制其临床应用的关键因素。本研究拟探讨黄芪桂枝五物汤对NSCLC伴LM患者培美曲塞鞘内化疗神经毒性的干预效果及安全性。

**方法:**

采用前瞻性、随机、双盲、对照设计纳入220例NSCLC伴LM患者，按照随机数字表法分为治疗组（黄芪桂枝五物汤+鞘内化疗）和对照组（模拟剂+鞘内化疗），每组110例。观察两组神经毒性发生率、毒性等级、疾病控制率（disease control rate, DCR）、无进展生存期（progression-free survival, PFS）、总生存期（overall survival, OS）、生活质量及不良反应。

**结果:**

治疗组神经毒性发生率为29.1%，显著低于对照组的51.8%（*P*<0.001）；治疗组1-2级和3-4级神经毒性发生率均低于对照组（*P*<0.010）；神经传导检测显示：治疗组腓肠神经感觉神经动作电位波幅高于对照组（*P*=0.012），正中神经、尺神经传导速度呈改善趋势。治疗组DCR为76.4%，高于对照组的69.1%（*P*=0.045）；治疗组中位PFS（7.8 *vs* 6.1个月，*P*<0.001）和OS（10.5 *vs* 8.9个月，*P*=0.007）均进一步延长。治疗组生活质量及中医证候评分改善高于对照组（*P*<0.05），两组不良反应发生率相当。

**结论:**

黄芪桂枝五物汤可有效降低NSCLC伴LM患者培美曲塞鞘内化疗所致神经毒性，提高化疗耐受性和生存获益，安全性良好。

## Abstract (en)

**Background and objective:**

Leptomeningeal metastasis (LM) is a severe complication of non-small cell lung cancer (NSCLC). Although intrathecal Pemetrexed chemotherapy can effectively prolong patient survival, a high incidence of neurotoxicity has emerged as a critical factor limiting its clinical application. This study aims to investigate the efficacy and safety of Huangqi Guizhi Wuwu decoction (HGWD) in mitigating neurotoxicity induced by intrathecal Pemetrexed chemotherapy in NSCLC patients with LM.

**Methods:**

In a prospective, randomized, double-blind, controlled trial, 220 NSCLC patients with LM were enrolled and randomly assigned to either the treatment group (HGWD+intrathecal chemotherapy) or the control group (placebo+intrathecal chemotherapy), with 110 patients in each group. The incidence and grade of neurotoxicity, disease control rate (DCR), progression-free survival (PFS), overall survival (OS), quality of life, and adverse reactions were observed and compared between the two groups.

**Results:**

The incidence of neurotoxicity in the treatment group was 29.1%, significantly lower than that in the control group (51.8%)(*P*<0.001). The incidences of both grade 1-2 and grade 3-4 neurotoxicity were lower in the treatment group than in the control group (*P*<0.010). Nerve conduction studies showed that the sensory nerve action potential amplitude of the sural nerve was significantly higher in the treatment group than in the control group (*P*=0.012), and the conduction velocities of the median and ulnar nerves showed a trend toward improvement. The DCR was 76.4% in the treatment group, higher than 69.1% in the control group (*P*=0.045). Both median PFS (7.8 *vs* 6.1 mon, *P*<0.001) and median OS (10.5 *vs* 8.9 mon, *P*=0.007) were significantly prolonged in the treatment group. Improvements in quality of life and traditional Chinese medicine syndrome scores were superior in the treatment group (*P*<0.05). No significant difference in adverse reactions were observed.

**Conclusion:**

HGWD can significantly reduce the incidence of neurotoxicity induced by Pemetrexed intrathecal chemotherapy, improve chemotherapy tolerance and survival benefits, with a good safety profile in NSCLC patients with LM.

软脑膜转移（leptomeningeal metastasis, LM）是非小细胞肺癌（non-small cell lung cancer, NSCLC）最为严重的并发症之一，患者预后极差，中位生存期仅3个月左右^[1,2]^。由于血脑屏障的限制，传统静脉化疗及靶向药物难以有效渗透至脑脊液，无法在脑部组织中实现优化的治疗浓度，临床疗效受到制约^[3]^。近年来，临床研究^[4,5]^表明，培美曲塞鞘内化疗能够突破血脑屏障，实现药物在脑脊液中的高浓度分布，从而显著延长患者的生存期，显示出良好的应用前景。然而，值得注意的是，鞘内化疗所致神经毒性的发生率高达35%-55%，已成为阻碍该治疗方案在临床上进一步推广的关键因素^[6]^。目前，针对此类神经毒性，现代医学尚缺乏明确有效的预防与治疗手段^[7]^，因此探寻安全有效的干预策略具有重要临床意义。

中药复方是延续我国中医药体系完整性和可持续性的重要组成部分，其在肿瘤治疗中具有“减毒增效”的独特优势。黄芪桂枝五物汤出自《金匮要略》，为治疗“血痹”病症的经典方剂，其核心功效在于温补阳气、调和营卫以通经络，其组方与现代医学缓解神经病理性疼痛、改善微循环的病理环节具有高度相关性。基础研究^[8,9]^证实，该方可下调与痛觉传导密切相关的瞬时受体电位（transient receptor potential, TRP）通道家族的过度表达，从而减轻化疗神经毒性动物模型的行为学异常与神经组织损伤。此外，小样本临床观察^[10]^也提示，黄芪桂枝五物汤能够有效降低奥沙利铂等化疗药物所致周围神经毒性的发生率与严重程度，提高患者生存率。然而，该方针对培美曲塞鞘内化疗这一特殊给药途径所诱发的神经毒性是否具有确切的防治作用，目前尚缺乏高质量临床研究证据的支持。

基于以上背景，本研究依据中医“异病同治”理论，将经典方剂黄芪桂枝五物汤的创新性应用于防治鞘内化疗神经毒性这一新领域，首次设计并开展了一项前瞻性、随机对照临床研究（注册号：ChiCTR2400080702），旨在系统评价黄芪桂枝五物汤对NSCLC伴LM患者接受培美曲塞鞘内化疗期间神经毒性的干预效果与安全性。研究结果将有力验证中药对于鞘内化疗神经毒性的防治作用，为临床推广应用提供更高级别的循证依据，同时建立体现协同互补、高效低毒理念的“鞘内化疗+中药治疗+异病同治”的中西医结合肿瘤治疗模式，为丰富肿瘤临床治疗方案、减少毒副反应、提高临床疗效等提供理论和实践支撑。

## 1 资料与方法

### 1.1 一般资料

选取2023年1月至2025年6月于南京大学医学院附属鼓楼医院就诊并符合研究纳入标准的NSCLC伴LM患者作为研究对象。样本量计算：基于前期应用及文献报道^[6]^，对照组培美曲塞鞘内化疗神经毒性发生率约为50%。假设黄芪桂枝五物汤可将神经毒性发生率降低至30%（绝对获益20%），设α=0.05（双侧），检验效能1-β=0.80，采用两组独立样本率比较的公式计算，每组需样本量100例。考虑10%的脱落/失访率，最终确定每组纳入110例，总样本量220例。由不参与临床评估的独立统计人员通过计算机生成随机序列，将编号对应分配给外观完全一致的治疗颗粒剂和模拟剂；患者、临床医生及结局评估者均对分组不知情。按1:1分配至治疗组与对照组，每组各110例。两组患者在年龄、性别、美国东部肿瘤协作组体能状态（Eastern Cooperative Oncology Group performance status, ECOG PS）评分、表皮生长因子受体（epidermal growth factor receptor, *EGFR*）突变状态、既往治疗线数、糖尿病史、既往神经毒性药物暴露史等统计分析表明，各组间差异均未达到显著性水平（*P*>0.05），体现了样本的均衡性与可比性（表1）。所有受试者或其法定监护人均在研究启动前签署了知情同意书，确保临床与影像数据可用于学术研究。本研究方案已获南京大学医学院附属鼓楼医院伦理委员会批准并通过审核（伦理批号：2023-562-02），研究过程遵循《赫尔辛基宣言》、中国药品临床试验管理规范（Good Clinical Practice, GCP）指导原则、世界卫生组织GCP指导原则和人用药品注册技术国际协调会议GCP指导原则。

**表1:** 患者基线特征比较

Characteristic	Treatment group (*n*=110)	Control group (*n*=110)	*P*
Age (yr)	68.3±5.2	67.9±5.6	0.512
Gender			0.623
Male	67 (60.9%)	65 (59.1%)	
Female	43 (39.1%)	45 (40.9%)	
ECOG PS score			0.974
0	34 (30.9%)	32 (29.1%)	
1	48 (43.6%)	47 (42.7%)	
2	18 (16.4%)	20 (18.2%)	
3	10 (9.1%)	11 (10.0%)	
EGFR mutation status			0.621
Mutant	72 (65.5%)	68 (61.8%)	
Wild-type	38 (34.5%)	42 (38.2%)	
Prior lines of therapy			0.643
1	63 (57.3%)	60 (54.5%)	
≥2	47 (42.7%)	50 (45.5%)	
Diabetes history			0.856
Yes	18 (16.4%)	20 (18.2%)	
No	92 (83.6%)	90 (81.8%)	
Prior neurotoxic chemotherapy			0.891
Yes	42 (38.2%)	44 (40.0%)	
No	68 (61.8%)	66 (60.0%)	

ECOG PS: Eastern Cooperative Oncology Group performance status; EGFR: epidermal growth factor receptor.

### 1.2 纳入与排除标准

（1）纳入标准：①年龄18-75岁（覆盖肺癌LM的主要发病人群；75岁以上患者因合并症多、药物代谢差异大，已另行开展专项研究）；②经病理确诊为肺腺癌，且经临床病理或影像学评估确诊为LM；③ECOG PS评分≤3分；④血液学及生化指标符合化疗要求；⑤签署知情同意书。（2）排除标准：①肺鳞癌或小细胞癌；②对培美曲塞过敏；③近期接受其他抗肿瘤治疗；④合并其他肿瘤或严重心、肝、肾功能异常及出血倾向；⑤研究者认为不适合入组者。

### 1.3 治疗方法

（1）研究用药名称和规格：①注射用培美曲塞：商品名普来乐，0.2 g/瓶，江苏豪森公司，30 ^o^C以下储藏保存，遮光密封，有效期18个月。②黄芪桂枝五物汤：颗粒剂，12 g/袋，30 ^o^C以下储藏保存，遮光密封，有效期12个月。本品含芍药苷、毛蕊异黄酮葡萄糖苷、甘草苷及桂皮醛的含量分别不少于7.9、0.2、4.7、30.2 mg/g。③黄芪桂枝五物汤模拟剂：定制的外观、气味、包装均与治疗组颗粒剂一致的模拟剂（不含有效药物成分），12 g/袋，30 ^o^C以下储藏保存，遮光密封，有效期12个月。（2）治疗方案：对照组：经腰椎穿刺规律行培美曲塞（30 mg/m²）鞘内化疗，于d1、d8给药，21 d为1个周期，共4个周期；同时口服黄芪桂枝五物汤模拟剂，1袋/次，2次/d，持续至全部鞘内化疗结束后1周。治疗组：鞘内化疗方案同对照组；口服黄芪桂枝五物汤颗粒剂，1袋/次，2次/d，持续至全部鞘内化疗结束后1周。

所有患者在研究期间，除方案规定的干预措施外，均不得使用其他具有明确神经保护作用或治疗神经病变的药物。两组均给予相同的常规对症支持治疗及预防性用药（如护胃、止吐、水化、抗过敏等）。

### 1.4 观察指标

#### 1.4.1 主要终点

神经毒性发生率与严重程度：（1）发生率：计算每组发生治疗相关神经毒性（包括中枢性与外周性）的患者比例。（2）严重程度：采用美国国家癌症研究所不良事件通用术语评价标准（National Cancer Institute-Common Terminology Criteria for Adverse Events, NCI-CTCAE）5.0版进行分级评估。于每个治疗周期结束后进行记录。重点关注≥3级严重神经毒性事件。（3）客观电生理评估：为提供客观量化指标，于基线及研究结束时，对所有患者进行神经传导检测。运用肌电图/诱发电位检测设备，对两侧正中神经、尺神经及腓肠神经进行运动神经传导速度、感觉神经传导速度、复合肌肉动作电位时程以及感觉神经动作电位波幅的评估分析，以评估外周神经功能的实际变化。

#### 1.4.2 次要终点

（1）鞘内化疗抗肿瘤疗效：采用神经肿瘤反应评估标准（Response Assessment in Neuro-oncology, RANO）^[11]^，通过头颅及全脊柱磁共振成像（magenetic resonance image, MRI）（平扫+增强，黑血序列）影像学检查和神经系统临床表现，综合评价LM的病情变化。疗效分为完全缓解、部分缓解、疾病稳定和疾病进展。疾病控制率（disease control rate, DCR）计算为达到完全缓解、部分缓解和疾病稳定的患者占总人群的百分比。评估时间点为每2个治疗周期结束后3周。（2）生存预后：无进展生存期（progression-free survival, PFS）：从随机入组日期到首次经影像学或临床证实为疾病进展，或因任何原因死亡的日期。总生存期（overall survival, OS）：从随机入组日期到因任何原因导致死亡的日期。研究结束时仍未死亡或失访的患者，其数据将在末次随访日期（2025年12月31日）进行截尾。（3）生活质量：采用欧洲癌症研究与治疗组织生命质量核心量表（European Organization for Research and Treatment quality of life questionnare-core 30, EORTC QLQ-C30）中文版进行评估。该量表由5个功能维度（躯体、角色、认知、情绪、社会功能）、3个症状维度（疲劳、疼痛、恶心呕吐）、1个整体生活质量维度以及若干单项测量条目构成。在基线期和治疗结束后，由经过统一培训的研究人员指导患者完成量表填写，通过比较各维度得分的变化，评估患者生活质量的改善情况。（4）中医证候积分：参照《中药新药临床研究指导原则》及“血痹”证候特点，制定中医证候评分表。对肢体麻木、疼痛、感觉异常、恶风畏寒、少气懒言、神疲倦怠等主次症状进行量化评分（0分：无症状；1分：轻度；2分：中度；3分：重度）。由对分组不知情的独立中医师进行评估，确保盲法实施。计算总积分，于基线及每个治疗周期结束后进行评估。

#### 1.4.3 安全性指标

全面记录研究期间发生的所有不良事件，无论是否与研究治疗相关，均参照NCI-CTCAE 5.0标准进行严重程度分级。定期监测血常规、肝肾功能、凝血功能等实验室指标。特别关注与黄芪桂枝五物汤潜在相关的不良反应，如胃肠道不适、过敏反应等。建立独立的数据安全监察委员会，对所有严重不良事件及非预期事件进行审阅与评估，确保受试者安全。

### 1.5 统计学方法

采用SPSS 22.0软件进行数据统计分析。计量资料经正态性检验，不符合正态分布者以中位数（第25百分位数，第75百分位数）[M (P25, P75)]表示，组间比较采用*Mann-Whitney U*检验，结果以*Z*值表示；符合正态分布者以均数±标准差（Mean±SD）表示，组间比较采用*t*检验。计数资料以例数（构成比）[*n*(%)]表示，并通过χ²检验评估组间差异。采用*Kaplan-Meier*法绘制生存曲线，组间比较采用*Log-rank*检验。*P*<0.05为差异具有统计学意义。

## 2 结果

### 2.1 神经毒性发生率比较

根据NCI-CTCAE 5.0标准评估，治疗组神经毒性总发生率为29.1%（32/110），明显低于对照组的51.8%（57/110），差异具有统计学意义（*P*<0.001）。进一步分析神经毒性分级分布情况显示，治疗组1-2级神经毒性发生率为25.5%（28/110），显著低于对照组的39.1%（43/110）（*P*=0.008）；而3-4级神经毒性发生率在治疗组中为3.6%（4/110），亦显著低于对照组的12.7%（14/110）（*P*=0.001）。神经传导检测结果显示：治疗组腓肠神经感觉神经动作电位波幅高于对照组[(8.3±2.1) *vs* (6.1±1.9) μV, *P*=0.012]；正中神经感觉传导速度[(52.4±5.3) *vs* (50.1±5.8) m/s, *P*=0.086]及尺神经感觉传导速度[(51.7±5.1) *vs* (49.8±5.6) m/s, *P*=0.094]组间差异无统计学意义。结果表明，黄芪桂枝五物汤不仅显著降低了总体神经毒性发生率，亦有效减轻了神经毒性的严重程度。

### 2.2 临床疗效与生存分析比较

根据RANO标准评估，治疗组DCR为76.4%（84/110），高于对照组的69.1%（76/110），差异有统计学意义（*P*=0.045）。生存分析结果进一步显示，治疗组中位PFS为7.8个月，显著长于对照组的6.1个月（*P*<0.001）；治疗组中位OS为10.5个月，亦显著长于对照组的8.9个月（*P*=0.007）。*Kaplan-Meier*生存曲线见图1。

**图1:**
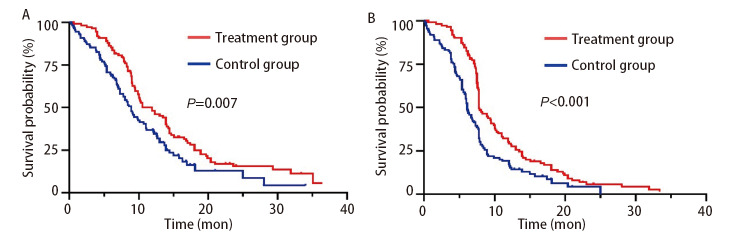
两组患者OS与PFS的Kaplan-Meier曲线。A：OS曲线，治疗组中位OS为10.5个月，对照组为8.9个月（P=0.007）；B：PFS曲线，治疗组中位PFS为7.8个月，对照组为6.1个月（P<0.001）。

### 2.3 生活质量及中医证候评分比较

经治疗后，治疗组在生活质量及由独立中医师（盲法）评估的中医证候评分方面均表现出显著改善（表2）。通过EORTC QLQ-C30 量表评估，治疗组在整体生活质量（*P*=0.011）、角色功能（*P*=0.023）和情绪功能（*P*=0.016）领域的评分提升均显著优于对照组。同时，治疗组在疼痛（*P*=0.008）和失眠（*P*=0.019）等症状领域的改善也更为明显。中医证候评分方面，治疗组在肢体麻木（*P*<0.001）、疼痛（*P*=0.002）、感觉异常（*P*=0.003）等“血痹”相关核心症状的积分下降幅度显著大于对照组，总体证候改善有效率为78.2%，显著高于对照组的58.2%（*P*<0.001）。

**表2:** 两组患者生活质量及中医证候评分比较 [M (P_25_, P_75_)]

Item	Treatment group (*n*=110)	Control group (*n*=110)	Z/χ²	*P*
EORTC QLQ-C30 functional scales				
Global health status	8.5 (5.0, 12.0)	4.0 (0.0, 8.0)	-2.541	0.011
Role functioning	9.5 (5.0, 14.0)	5.0 (0.0, 10.0)	-2.278	0.023
Emotional functioning	9.0 (5.0, 13.0)	5.0 (0.0, 9.0)	-2.413	0.016
EORTC QLQ-C30 symptom scales				
Pain	-10.0 (-15.0, -5.0)	-5.0 (-10.0, 0.0)	-2.658	0.008
Insomnia	-10.0 (-15.0, -5.0)	-5.0 (-10.0, 0.0)	-2.351	0.019
Traditional Chinese medicine syndrome scores				
Numbness of limbs	-1.0 (-2.0, -1.0)	-0.5 (-1.0, 0.0)	-3.862	<0.001
Pain	-1.0 (-1.0, 0.0)	-0.5 (-1.0, 0.0)	-3.124	0.002
Paresthesia	-1.0 (-2.0, -0.5)	-0.5 (-1.0, 0.0)	-2.987	0.003
Total syndrome score	-5.0 (-7.0, -3.0)	-2.5 (-4.0, -1.0)	-4.126	<0.001
ORR of syndrome [n (%)]	86 (78.2)	64 (58.2)	8.547*	<0.001

*: χ² test for categorical variable (overall response rate of syndrome); Mann-Whitney U test for continuous variables (Z values reported). For functional scales of EORTC QLQ-C30, higher scores indicate better functioning. Symptom scales and Traditional Chinese medicine syndrome scores are presented as changes from baseline, with negative values indicating improvement. EORTC QLQ-C30: European Organization for Research and Treatment quality of life questionnare-core 30; ORR: overall response rate.

### 2.4 安全性比较

在安全性方面，两组治疗相关不良事件总体发生率相近，差异无统计学意义（*P*>0.05）。最常见的不良事件为1-2级胃肠道反应（治疗组 *vs* 对照组：11.8% *vs* 10.9%）及轻度肝功能异常（治疗组 *vs* 对照组：8.2% *vs* 9.1%）。所有不良事件经对症处理后均缓解，未导致治疗中断。研究期间未发生与黄芪桂枝五物汤相关的严重不良事件或非预期严重反应。

## 3 讨论

培美曲塞是一种多靶点抗叶酸剂，于2017年由国家食品药品监督管理局批准上市，对NSCLC具有良好的抗肿瘤活性^[12]^。前期研究^[4,5]^表明，培美曲塞鞘内化疗对难治性LM疗效显著。但相关研究^[6,13]^显示，培美曲塞鞘内化疗可导致35%-55%的患者发生神经毒性，其主要包括中枢和外周神经毒性。中枢神经毒性是指接受鞘内化疗的肿瘤患者出现化疗相关认知障碍（也称“化疗脑”）、脑病、头痛或癫痫发作等。外周神经毒性反应常见的是神经痛和感觉障碍（或异常）等外周神经功能紊乱症状，与外周神经损害直接相关。神经毒性是培美曲塞鞘内化疗的主要剂量限制性毒性，主要参照其他病因如物理化学、糖尿病等导致的神经病变的治疗方案进行处理。临床多采用神经营养剂甲钴胺、抗氧化剂谷胱甘肽、维生素类药物、德巴金等抗抑郁或抗癫痫药，其治疗有效率和证据级别均不高^[14]^，且目前对神经毒性不存在可供推荐的预防方案^[7]^。因此，寻找预防或治疗鞘内化疗神经毒性的药物将具有重大的临床意义和社会价值，对患者能够积极接受鞘内治疗、减少不良反应、提高患者生活质量并最终改善预后有着重要的现实意义。

培美曲塞鞘内化疗所致神经毒性归属于祖国医学“痹证”“血痹”的范畴。中医认为化疗药多苦寒，使机体气血亏虚、经脉寒凝、脉络痹阻，故临床治疗方案多遵循益气养血、温经通络、温阳散寒等。黄芪桂枝五物汤是治疗血痹症的经典名方，该方剂主要由黄芪、白芍、桂枝、生姜及大枣五味药材构成。其中黄芪作为君药，具有补益元气与驱邪外出的功效；桂枝配合黄芪使用，既可温通经脉又助益气温阳之效，同时兼有活血化瘀之功的芍药养血活血，化癖通络，与桂枝相伍，共为臣药；生姜因性温味辛，能发散风寒、温经通脉，故为佐药以增强桂枝的温经散寒之效；大枣性平味甘，既能调和诸药，又与生姜相配协同桂枝、白芍调和营卫，发挥重要的辅助作用。诸药相伍，共收益气通经、活血蠲痹之效。现代药理研究^[10]^表明，黄芪桂枝五物汤能够显著改善受损区域的微循环状态，促进周围神经损伤后施万细胞增殖，保护神经元功能，并加速神经修复与结构重塑过程。

研究^[15]^证实多种thermo-TRPs的异常表达和激活与神经毒性的发生密切相关。TRPs是位于细胞膜上的一组重要的非选择性阳离子通道^[16]^，thermo-TRPs作为环境感受器，能广泛响应包括温度、机械张力及pH在内的物理化学刺激。更重要的是，其在病理条件下通过介导痛觉信号通路，直接参与并诱发了神经病理性疼痛的形成过程^[17]^。多种thermo-TRPs是化疗药物作用于感觉神经系统的重要靶点，其被激活后可引发神经元的异常兴奋与损伤级联反应，从而导致神经毒性发生^[18]^。黄芪桂枝五物汤可显著下调痛觉感受相关TRPs离子通道蛋白TRPM8、TRPV1、TRPA1 mRNA表达。电生理实验结果^[8,9]^表明，黄芪桂枝五物汤主要成分桂皮醛、姜烯酚对TRPs通道有调节作用。以上结果从thermo-TRPs视角初步揭示了黄芪桂枝五物汤干预化疗神经毒性的可能调控机制。另有研究^[19]^发现，通过灌胃给予大鼠黄芪桂枝五物汤提取液后，其机械性疼痛耐受阈值与冷觉痛阈显著升高，同时背根神经节神经元损伤程度明显减轻，进一步佐证了该方剂在缓解化疗引发的神经毒性方面的潜在疗效。本研究观察到神经毒性显著降低，结合前期基础研究，我们推测黄芪桂枝五物汤可能通过调节TRPV1/TRPA1通道活性，从而抑制培美曲塞引发的神经病理性疼痛信号传导。但上述机制与临床结果的直接关联尚不明确，未来需检测患者脑脊液中神经损伤标志物或TRPs通道相关信号分子以进一步验证。

本研究是首个针对黄芪桂枝五物汤防治培美曲塞鞘内化疗神经毒性的前瞻性随机对照临床研究。结果表明，在标准鞘内化疗基础上联用黄芪桂枝五物汤，能将神经毒性发生率从51.8%显著降低至29.1%，并进一步延长患者的PFS和OS，且安全性良好。这为解决鞘内化疗的剂量限制性毒性这一关键临床难题，提供了行之有效的中西医结合解决方案。研究创新点在于将中医“异病同治”法则成功应用于全新的临床场景。尽管该方最初在奥沙利铂等铂类药物导致的周围神经毒性中显示出疗效^[10]^，但我们基于“血痹”这一共同的核心病机，将其拓展应用于鞘内化疗这一特殊给药方式引起的中枢及外周神经毒性，并获得了高级别循证医学证据的支持。这不仅验证了中医理论的科学性与实用性，也为经典名方的现代化研究与临床应用开辟了新路径。本研究也存在一定的局限性：DCR 7.3%的绝对获益虽具统计学差异，但其临床显著性尚需多中心大样本研究进一步验证；中药复方具有多成分、多靶点的作用特点，其完整的药效物质基础及精确的分子机制网络仍有待后续基础研究的进一步揭示。

综上所述，本研究证实了黄芪桂枝五物汤能安全有效地降低NSCLC伴LM患者培美曲塞鞘内化疗的神经毒性，并带来生存获益。下一步的研究方向包括开展更大样本的多中心临床验证、深入探究其作用机制以及进行剂型优化以提高患者用药的依从性。
